# GSTT1 Deletion Is Related to Polycyclic Aromatic Hydrocarbons-Induced DNA Damage and Lymphoma Progression

**DOI:** 10.1371/journal.pone.0089302

**Published:** 2014-02-20

**Authors:** Fan Yang, Jie Xiong, Xiao-E Jia, Zhao-Hui Gu, Jing-Yi Shi, Yan Zhao, Jun-Min Li, Sai-Juan Chen, Wei-Li Zhao

**Affiliations:** 1 State Key Laboratory of Medical Genomics, Shanghai Institute of Hematology, Shanghai Rui Jin Hospital, Shanghai Jiao Tong University School of Medicine, Shanghai, China; 2 Pôle de Recherches Sino-Français en Science du Vivant et Génomique, Laboratory of Molecular Pathology, Shanghai, China; 3 Institute of Health Sciences, Shanghai Institutes for Biological Sciences and Graduate University, Chinese Academy of Sciences & Shanghai Jiao Tong University School of Medicine, Shanghai, China; 4 Shanghai Centre for Systems biomedicine, Shanghai Jiao Tong University, Shanghai, China; University of Nebraska - Lincoln, United States of America

## Abstract

The interrelationship between genetic susceptibility and carcinogenic exposure is important in cancer development. Polymorphisms in detoxification enzymes of the glutathione-S-transferases (GST) family are associated with an increased incidence of lymphoma. Here we investigated the molecular connection of the genetic polymorphism of *GSTT1* to the response of lymphocytes to polycyclic aromatic hydrocarbons (PAH). In neoplastic situation, *GSTT1* deletions were more frequently observed in lymphoma patients (54.9%) than in normal controls (42.0%, P = 0.009), resulting in an increased risk for lymphoma in individuals with *GSTT1*-null genotype (Odds ratio = 1.698, 95% confidence interval = 1.145–2.518). *GSTT1* gene and protein expression were accordingly decreased in *GSTT1*-deleting patients, consistent with activated profile of cell cycle regulation genes. Mimicking environmental exposure using long-term repeat culture with low-dose PAH metabolite Hydroquinone, malignant B- and T-lymphocytes presented increased DNA damage, pCHK1/MYC expression and cell proliferation, which were counteracted by ectopic expression of *GSTT1*. Moreover, *GSTT1* expression retarded xenograft tumor formation of Hydroquinone-treated lymphoma cells in nude mice. In non-neoplastic situation, when zebrafish was exposed to PAH Benzo(a)pyrene, molecular silencing of *gstt1* enhanced the proliferation of normal lymphocytes and upregulated *myca* expression. Collectively, these findings suggested that *GSTT1* deletion is related to genetic predisposition to lymphoma, particularly interacting with environmental pollutants containing PAH.

## Introduction

During the past decades, the incidence of lymphoma has been significantly increased, ranging it among the ten most frequent cancers [1]. The etiologies of lymphoma remain largely undetermined. However, epidemiological studies revealed that exposure to environmental pollutants is a susceptibility factor [2]. Polycyclic aromatic hydrocarbons (PAH) represent the main components of environmental pollutants that have genotoxic and carcinogenic properties.

Genetic polymorphisms in detoxification enzymes are important determinants of individual variation in cancer risk. Glutathione S-transferases (GST) are the major detoxification enzymes in humans. As phase II biotransformation enzymes, GST catalyze the conjugation of reduced glutathione to electrophilic centres on a wide range of substrates, including activated exogenous molecules like PAH.

Several GST polymorphisms commonly occurring in humans are associated with an increased susceptibility to cancers, when combined with environmental factors. Recently, the role of GST genotypes in the pathogenesis of lymphoma has been addressed [3]. GSTT1 is an important member of GST family and involved in the detoxification of various carcinogens, particularly PAH. Evidence of an elevated risk for lymphoma in individuals with *GSTT1*-null homozygotes has been reported [4], [5], [6]. Proposed reasons could include an impaired neutralization of reactive oxygen species or reduced deactivation of carcinogenic intermediates of PAH. However, the exact molecular connection between *GSTT1* deletions and lymphoma development remained to be investigated.

In the present study, we examined the genetic polymorphisms of *GSTT1* in Chinese patients with lymphoma in comparison with a health control cohort, correlating the *GSTT1*-null genotype with the progression of lymphoma cells and the proliferative behavior of normal lymphocytes under the exposure of PAH. Our results showed that *GSTT1* deletion could be a potential risk factor of lymphomagenesis. Genetic susceptibility may interact with the genotoxic effect of environmental carcinogens to eventually predispose to lymphoma.

## Patients and Methods

### Ethics Statement

Written informed consent was obtained from all the patients (from the next of kin, caretakers, or guardians on the behalf of the minors/children patients) in accordance with the Declaration of Helsinki. The study was approved by the Shanghai Rui Jin Hospital Review Boards. Animals were used according to the protocols approved by the Shanghai Rui Jin Hospital Animal Care and Use Committee.

### Patients

Two hundred and four patients with lymphoma [103 diffuse large B-cell lymphoma (DLBCL) and 101 T-cell lymphoma (TCL) cases], 127 men and 77 women aged 14 to 82 years were included in this study. Histological diagnoses were established according to the World Health Organization classifications. Frozen tumor specimen was available in 114 lymphoma patients and 40 age- and sex-matched reactive hyperplasia cases.

### Cell Lines and Reagents

The B-lymphoma cell line Namalwa and T-lymphoma cell line Jurkat were obtained from American Type Culture Collection. Hydroquinone (Sigma-Aldrich) was dissolved in normal saline before use. Benzo(a)pyrene (BaP, Sigma-Aldrich) was dissolved in dimethyl sulfoxide (DMSO) as a stock solution of 400 mg/ml.

### Cell Proliferation and Flow Cytometric Assay

Cell growth was measured by MTT assay and cell proliferation was determined by 5-ethynyl-2′-deoxyuridine (EdU) incorporation assay using Cell-Light EdU kit (Ribobio Co., Ltd., China) at 48 h. Cell cycle and cell apoptosis were assessed at 48 h as previous reported [7].

### Genome-wide Copy Number Variation (CNV) Analysis

Genomic DNA was extracted using Wizard Genomic DNA Purification Kit (Promega). Genome-wide CNV genotyping was performed on frozen tumor samples of 25 DLBCL, 20 TCL and 8 reactive hyperplasia cases, using Human 610-Quad_v1 (610 k SNP probes) or 660 W-Quad_v1 (660 k SNP probes) DNA Analysis BeadChips. Regions were determined based on the Log R Ratio (LRR) of the signal intensity and B allele frequency (BAF) of genotyping call from the sample using platform of GenomeStudio V2011.1 with CnvPartition 3.1.6 (Illumina). All the data is available on NCBI (Accession number GSE47357).

### GSTT1 Genotyping and Expression

*GSTT1* deletion was detected on frozen tumor (114 cases) and peripheral blood (the rest 90 cases without frozen tumor specimen) of lymphoma patients by multiplex polymerase chain reaction (PCR) method, using albumin gene as an internal positive control, as previously reported [8]. The normal control group comprised 205 unrelated healthy volunteers. Blood samples were collected and leukocytes were isolated after hypotonic lysis of erythrocytes. Genomic DNA was extracted and amplified using the primers: *GSTT1*∶5′-TTCCTTACTGGTCCTCACATCTC-3′ and 5′-TCACCGGATCATGGCCAGCA-3′, and albumin:5′-GCCCTCTGCTAACAAGTCCTAC-3′ and 5′-GCCCTAAAAAGAAAATCGCCAATC-3′.

Total RNA was extracted using Trizol reagent and reverse-transcribed using PrimeScript RT reagent Kit with gDNA Eraser (TaKaRa). Real-time PCR was performed on frozen samples of lymphoma and reactive hyperplasia patients, using ABI PRISM 7900HT and specific probes for *GSTT1* (Assay ID: Hs01091675_g1) and *GAPDH* (Life Technologies). A relative quantification was calculated using the 2^−ΔΔCT^ method.

### Gene Network and Pathway Analysis

Human Genome U133 Plus 2.0 Array GeneChip microarray (Affymetrix) was performed on tumor samples of 8 DLBCL patients and analyzed by Expression Console software (Partek GS 6.5, Affymetrix). The data is available on NCBI (Accession number GES47355). Human LncRNA Microarray V2.0 (Arraystar Inc.) was performed on tumor samples of 8 TCL cases and analyzed by Agilent Feature Extraction Software (Agilent Technologies).

Genes were subsequently filtered by comparing their expression levels between the *GSTT1*-deleting and non-deleting patients. Statistical differences were calculated and the genes with *P*<0.05 were analyzed for enrichment of KEGG pathways using Database for Annotation, Visualization and Integrated Discovery (DAVID v6.7, http://david.abcc.ncifcrf.gov) for network composition analyses. Genes of pathway(s) significantly involved in both DLBCL and TCL were hierarchical clustered using MeV v4.8.1 (Dana-Farber Cancer Institute).

### GSTT1 Transfection

*GSTT1* expression vector (GSTT1, NM_000853.2) and the negative control vector (FU, pReciever-M46) were obtained from GeneCopoeia. The recombinant lentivirus vector PGC-GSTT1-IRES-GFP-LV and PGC-FU-GFP-LV were produced and packaged by co-transfecting 293T cells with the package vector. The supernatant of 293T cell culture were condensed and the virus titers were approximately 3×10^9^ TU/ml. To transfect Namalwa and Jurkat cells, the multiplicities of infection were 50 and 10, respectively.

### Comet Assay

DNA damage was determined by the comet assay with Reagent Kit for Single Cell Gel Electrophoresis Assay (Trevigen, Inc.). In addition to frank DNA strand breaks, oxidised bases were measured by conversion to breaks using endonuclease III (recognizing oxidised pyrimidines) or formamidopyrimidine DNA glycosylase (FPG, specific for oxidised purines). Measurements of comet parameters % DNA in the tail, tail length and tail moment were obtained. Net enzyme-sensitive sites were calculated by subtracting the comet score after incubation with buffer alone from the score with enzyme.

### Tissue Array

A human lymphoma tissue array (NHL482) was obtained from US Biomax, Inc. GSTT1 and p53-binding protein 53 BP1 expression were scored semi-quantitatively based on staining intensity and distribution using the immunoreactive score, as previously reported [7].

### Immunohistochemistry and Immunofluorescence Assay

Immunohistochemical analyses were carried out on 5-µm-paraffin sections or acetone-fixed cells with an indirect immunoperoxidase method using the antibodies against GSTT1, 53 BP1 (Abcam), Ki67 (Dako) and MYC (Abcam). Immunofluorescence assay was performed on acetone-fixed cells using mouse anti-human-γH2AX antibody followed by donkey anti-mouse-IgG antibody, as well as rabbit anti-human-53 BP1 and rabbit anti-human-pCHK1 antibody followed by donkey anti-rabbit-IgG antibody (Abcam). Nuclei were counterstained with DAPI.

### Western Blot

Western blot was performed as previously described [7]. Actin (Sigma) and LaminB (Abcam) were used to ensure equivalent loading of total and nuclear protein, respectively. Antibodies against GSTT1 and CHK1 were obtained from EPITOMICS. Anti-pCHK1, pCHK2 and CHK2 antibodies were from Cell Signaling. Anti-MYC and 53 BP1 antibodies were from Abcam. Horseradish peroxidase-conjugated goat anti-mouse-IgG and goat anti-rabbit-IgG were from Santa Cruz Biotechnology Inc.

### Tumorigenicity Assay in Murine Models

Five-week-old female BALB/c nude mice were obtained from Shanghai Laboratory Animal Center. Mice were injected subcutaneously into the right flank with lymphoma cells. For each cell line, mice were divided into 3 subgroups. Namalwa cells were injected with 1×10^7^, 2×10^6^ and 5×10^5^, and Jurkat cells were injected with 4×10^7^, 1×10^7^ and 2×10^6^, respectively. The number of the tumors formed was determined until 4 weeks after injection. Then mice were sacrificed, with tumor tissue samples fixed in formaldehyde and further processed for paraffin embedding.

### Cloning and Plasmid Construction in Zebrafish

Adult zebrafish (*Danio rerio*) were maintained following established guidelines [9] at 28°C on a 14 h:10 h light:dark cycle. Zebrafish *gstt1a* and *gstt1b* genes were identified based on homology to human *GSTT1*. The specific primers were designed according to genomic sequence in the UCSC data base (University of California, Santa Cruz) to amplify part of *gstt1a* and *gstt1b* genes: *gstt1a*:5′- CGGGATCCGGCGCCTCTCTTCTTTTCTT-3′ and 5′-CCCTCGAG CTGCATCATCTCCACAATGG-3′; *gstt1b*:5′-CGGGATCCTGATCCCAAAGGGTTGAGTC-3′ and 5′-CCCTCGAGGAACAAAAAGGTGCCATTCG-3′; *gstt1a*-EGFP reporter:5′- GATCCACTTACTTCCACAATGCCGCTGGAGCTGTATCTCGATCTGCATTCCCAGCCG-3′ and 5′- AATTCGGCTGGGAATGCAGATCGAGATACAGCTCCAGCGGCATTGTGGAAGTAAGTG-3′; *gstt1b*-EGFP reporter:5′- GATCCCTTTTTAAACATGACTCTGGAAATTTACTTGGACCTGTTTTCCCAGCCCTGG-3′ and 5′- AATTCCAGGGCTGGGAAAACAGGTCCAAGTAAATTTCCAGAGTCATGTTTAAAAAGG-3′; *myca*:5′-CGGGATCCTTTCCAAGAACTCCCACCCC-3′ and 5′-CCCTCGAGTTTAATCTAGGGCTGCGCAG-3′. The amplified PCR product was purified and subcloned into pCS2^+^ for subsequent *in vitro* synthesis of mRNA.

### Whole-mount in situ Hybridization (WISH) in Zebrafish

To localize *gstt1a* and *gstt1b* in zebrafish, whole-mount *in situ* hybridization (WISH) was carried out. pCS2^+^ containing part of *gstt1a*, *gstt1b*, *rag1* or *myca* (729 bp, 473 bp, 1000 bp and 231 bp, respectively) was utilized to generate antisense RNA probes for *gstt1a*, *gstt1b*, *rag1* and *myca* using digoxigenin-11-uridine 5′-triphosphate (Roche Applied Science). Zebrafish embryos were fixed in 4% paraformaldehyde at the stages indicated and WISH was performed as previously described [10].

### Microinjection of Zebrafish Embryos

The *gstt1a* and *gstt1b* morpholino oligonucleotides (MO) and its 5 bp-mismatch control were synthesized by Gene Tools LLC. The sequences were as follows: *gstt1a*:5′-CGAGATACAGCTCCAGCGGCATTGT-3′; 5 bp-mismatch of *gstt1a*: 5′-CGAcATAgAcCTCCAcCcGCATTGT-3′; *gstt1b*:5′- GGTCCAAGTAAATTTCCAGAGTCAT-3′; 5 bp-mismatch of *gstt1b*:5′- GGTCgAAcTAAATTTgCAcAcTCAT -3′. MOs were diluted to 1 mM stock solution in 1×Danieau buffer and were microinjected at a volume of 2 nl into one-cell stage embryos using an air pressure injector and glass capillaries. Injection experiments were performed in triplicate.

The vectors pCS2^+^-MO-*gstt1a* and pCS2^+^-MO-*gstt1b* were linearized with XhoI and transcribed *in vitro* with SP6 RNA polymerase in the presence of m7G (59)ppp(59)G (Ambion) to produce capped transcripts and were microinjected into one-cell stage embryos for MO efficiency evaluation.

### Transmission Electron Microscopy

The embryos of zebrafish were fixed overnight with 2% glutaraldehyde/0.1 M phosphate-buffered saline (pH 7.3) at 4°C. The embryos were then post-fixed in 1% osmium tetroxide at 4°C for 1 h, rinsed thoroughly with distilled water, dehydrated by graded ethanol and freeze-dried. The samples were sputter-coated in Epon812 (TAAB Laboratories) and ultrathin sections were prepared, stained with uranyl acetate and lead citrate, and examined with a PhilipsCM120 transmission electron microscopy (Philips).

### Statistic Analysis

Assays were set up in triplicate and the results were presented as Mean±S.E. Variance between the experimental groups was determined by two-tailed t-test or Mann-Whitney test. The logistic regression test was used to compare the differences in genotype frequencies between patients and controls adjusted by sex and age. Differences were considered significant when the 2-sided *P*<0.05.

## Results

### GSTT1-null Genotype is Frequently Observed in Lymphoma Patients

**Figure 1:**
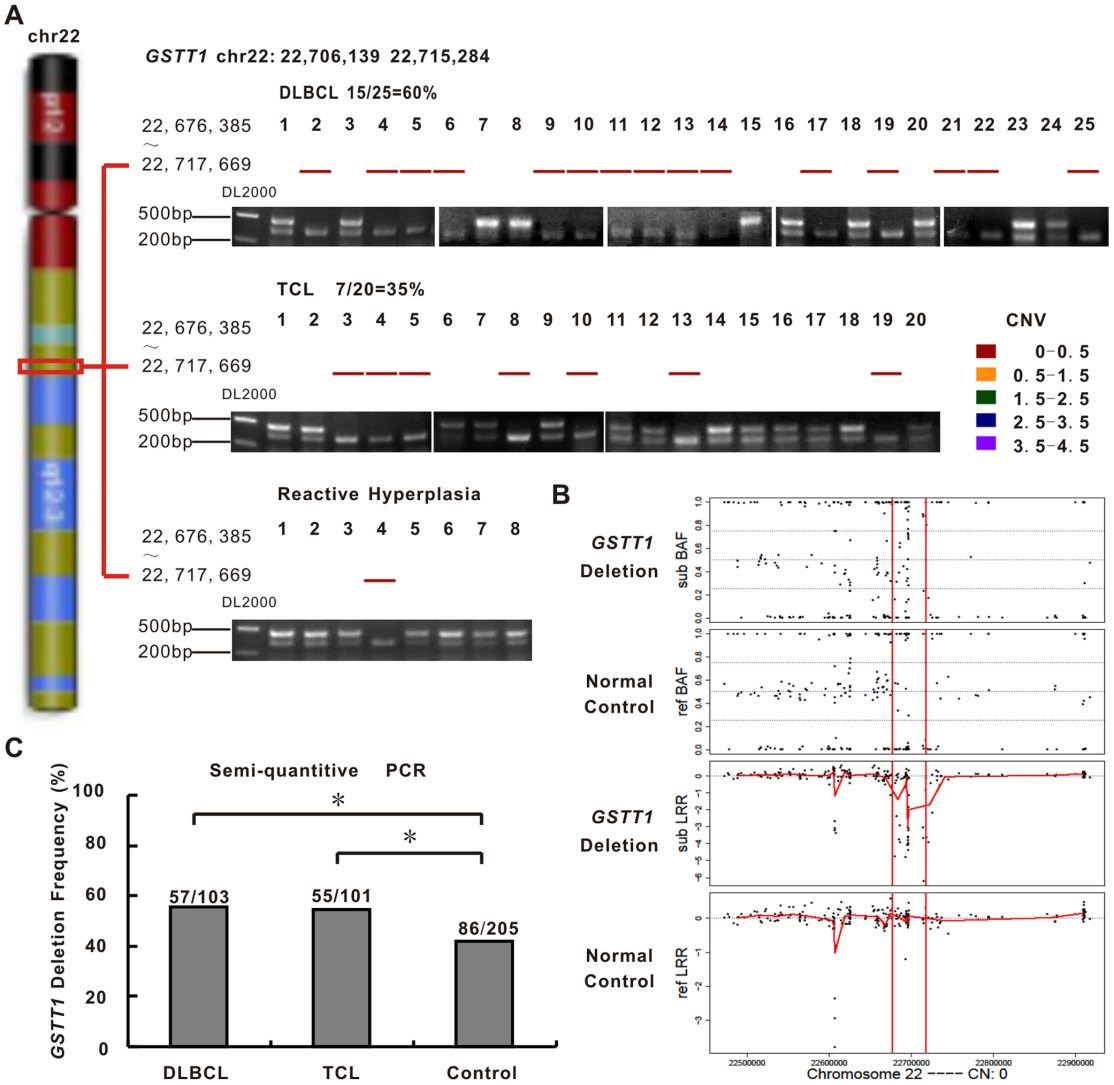
*GSTT1*-null genotype is frequently observed in patients with lymphoma. A: *GSTT1* deletion detected by copy number variation (CNV) analysis (upper panel) and validated by semi-quantitative PCR (lower panel) in diffuse large B-cell lymphoma (DLBCL, 15/25, 60.0%), T-cell lymphoma (TCL, 7/20, 35.0%) and reactive hyperplasia (1/8, 12.5%). B: SNP data illustrating the distribution of B allele frequencies (BAF) and Log R Ratio (LRR) values across the region of chromosome 22 (22,676,385 bp to 22,717,669 bp) in the *GSTT1*-deleting cases and normal controls. C: Semi-quantitative PCR validation of *GSTT1* deletion in expanded sample-set of DLBCL (57/103, 55.3%), TCL (55/101, 54.5%) and normal controls (86/205, 42.0%). *P<0.05 comparing with normal controls.

A homozygous loss in chr22q11.23 spanning *GSTT1* gene (22,706,139 bp to 22,715,284 bp, NCBI Build 36) was detected in 15/25 (60%) of DLBCL and 7/20 (35%) of TCL patients, comparing with 1/8 (12.5%) of reactive hyperplasia cases (Figure 1A). Figure 1B depicted data for the region of 22,500,000 bp to 22,900,000 bp of chromosome 22. In the normal control, the LRR was distributed around zero corresponding to DNA copy number 2, whilst the BAFs were clustered around values of 0, 0.5 and 1 that correspond to the diploid genotypes AA, AB and BB. The *GSTT1*-null genotype presented a much more complex scenario with extensive genomic rearrangements leading to considerable variation in the SNP data.

Using semi-quantitative PCR, *GSTT1* genotypes were further investigated. *GSTT1* gene deficiency was deduced from the absence of the specific 460 bp fragment. Presence of a 350 bp albumin fragment confirmed proper functioning of the method (Figure 1A). Of the 204 patients, 112 cases (54.9%) were *GSTT1*-null. Prevalence of the *GSTT1* deletions was 55.3% (57/103) in DLBCL and 54.5% (55/101) in TCL, significantly higher than that in normal controls (86/205, 42.0%, P = 0.027 and P = 0.034, respectively, Figure 1C), adjusted by sex and age. This translated into an increased risk of lymphoma for individuals with the *GSTT1*-null genotype (Odds ratio = 1.698; 95% confidence interval = 1.145–2.518).

### GSTT1 Deletion Results in Loss of Protein Expression

**Figure 2:**
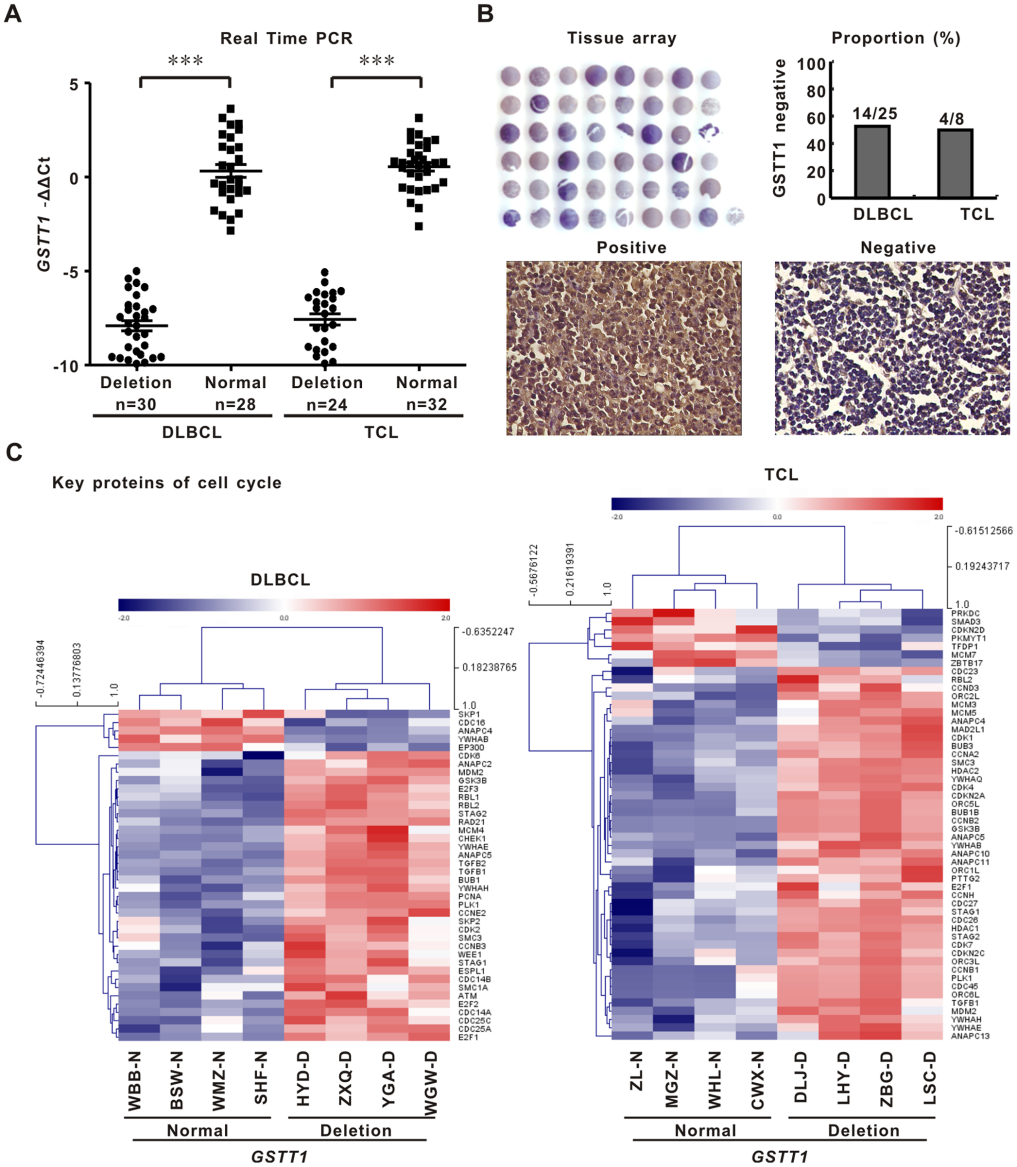
*GSTT1* deletion is related to decreased gene and protein expression and enhanced cell cycle progression. A: *GSTT1* gene expression assessed by quantitative real-time PCR in the *GSTT1*-deleting patients and normal controls. B: GSTT1 protein expression detected by tissue array. C: Geneset of cell cycle related proteins revealed by gene network and pathway analysis on microarray data of DLBCL and TCL.

*GSTT1* gene expression was measured by real-time quantitative PCR in 114 patients with frozen tissue specimen. *GSTT1*-deleting patients presented significant downregulation of *GSTT1* gene expression in both DLBCL and TCL, comparing with those without deletion (P both <0.001, Figure 2A). GSTT1 protein was detected by tissue array. GSTT1 was negative in 56.0% (14/25) of DLBCL and in 50.0% (4/8) of TCL, respectively, in accordance with the percentage of *GSTT1* deletion in the two subtypes (Figure 2B). Moreover, 53BP1 was more frequently expressed in GSTT1-negative cases than in GSTT1-positive cases [DLBCL, 85.7% (12/14) vs 45.5% (5/11), P = 0.032, T-NHL, 100% (4/4) vs 0% (0/4), P = 0.028].

Gene expression profile was assessed by microarray in frozen tissue sample of 8 DLBCL and 8 TCL patients with the history of PAH exposure (four each for *GSTT1*-deleting and non-deleting cases). The pathway identified with statistical significance by the differentially expressed *GSTT1* revolved around key proteins of cell cycle progression (Figure 2C), indicating that *GSTT1* deletion is biologically functional and relevant to lymphoma cell proliferation.

### GSTT1 Protects Lymphoma Cells from PAH-induced DNA Damage

**Figure 3:**
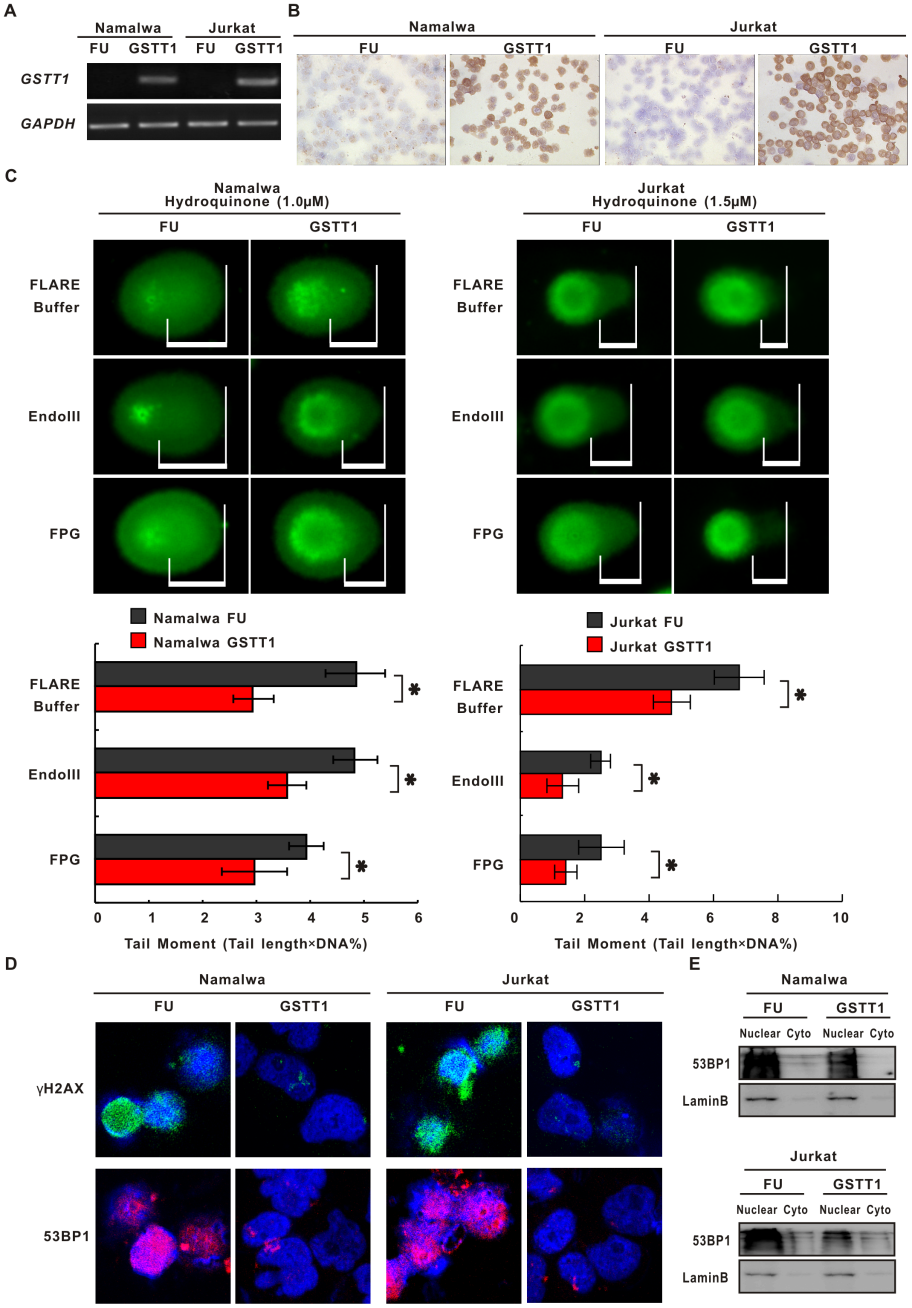
*GSTT1* expression protects lymphoma cells from PAH-induced DNA damage. A: *GSTT1* gene expression assessed by semi-quantitative PCR in Namalwa and Jurkat cells transfected with *GSTT1* (GSTT1) and the negative control vector (FU). B: Images represent results from three independent experiments. GSTT1 protein expression detected by immunohistochemistry assay. C: DNA damage measured by alkaline and modified comet assay in Namalwa and Jurkat cells treated with Hydroquinone (Upper panels). Mean tail moments were calculated in the same cells (Lower panels). Data represents Mean ± SE from at least 50 cells in each group. D: Immunofluorescence assay of γH2AX and 53BP1 in Hydroquinone-treated lymphoma cells. *P<0.05 comparing with the FU cells.

To further elucidate its biological function in lymphoma, *GSTT1* gene was transfected to *GSTT1*-negative Namalwa and Jurkat cells. Comparing with the negative control (FU), lymphoma cells expressing *GSTT1* (GSTT1) showed increased levels of gene and protein expression, as revealed by semi-quantitative PCR (Figure 3A) and by immunostaining assay (Figure 3B), respectively.

Hydroquinone, common metabolites of PAH [11], was used to treat lymphoma cells. Fifty percent of growth inhibition (IC50) was first measured in both cell lines at concentrations ranging from 1.25 to 40 µM. The IC50 value was 14.4 µM in Namalwa cells and 18.7 µM in Jurkat cells, respectively. To mimic the environmental exposure status, lymphoma cells were then cultured with low-dose Hydroquinone (at a concentration of approximately 10% of IC50 with minimal cytotoxicity, 1 µM for Namalwa GSTT1/FU cells and 1.5 µM for Jurkat GSTT1/FU cells, respectively) in a repeat manner [12]. Briefly, cells were treated once a week with Hydroquinone for 24 h and weekly for 4 times in total. Normal saline was referred as the solvent control.

DNA strand breaks were assessed using comet assay, and in addition DNA oxidation damage, namely oxidised pyrimidines and purines, using lesion specific enzymes Endo III and FPG. As manifested by increased parameters % DNA in the tail and prolonged tail length, DNA damage was less intensive in Hydroquinone-treated *GSTT1*-expressing lymphoma cells (GSTT1) than in the negative control cells (FU). Tail moment was further assessed by parameters % DNA×tail length. The results showed that tail moment, as well as EndoIII- and FPG-sensitive sites, were significantly reduced in the GSTT1 groups than in the FU groups (Namalwa, P = 0.037, P = 0.042 and P = 0.042, Jurkat, P = 0.041, P = 0.049 and P = 0.049, respectively, Figure 3C).

γH2AX and 53 BP1 are sensitive markers of DNA damage [13]. As detected by immunofluorescence assay, FU cells presented increased nuclear levels of γH2AX and 53 BP1, which were significantly prohibited in GSTT1 cells (Figure 3D). These data indicated that DNA damage is constitutively activated in response to PAH and could be protected by *GSTT1* expression.

### GSTT1 Inhibits PAH-mediated Lymphoma Cell Proliferation

**Figure 4:**
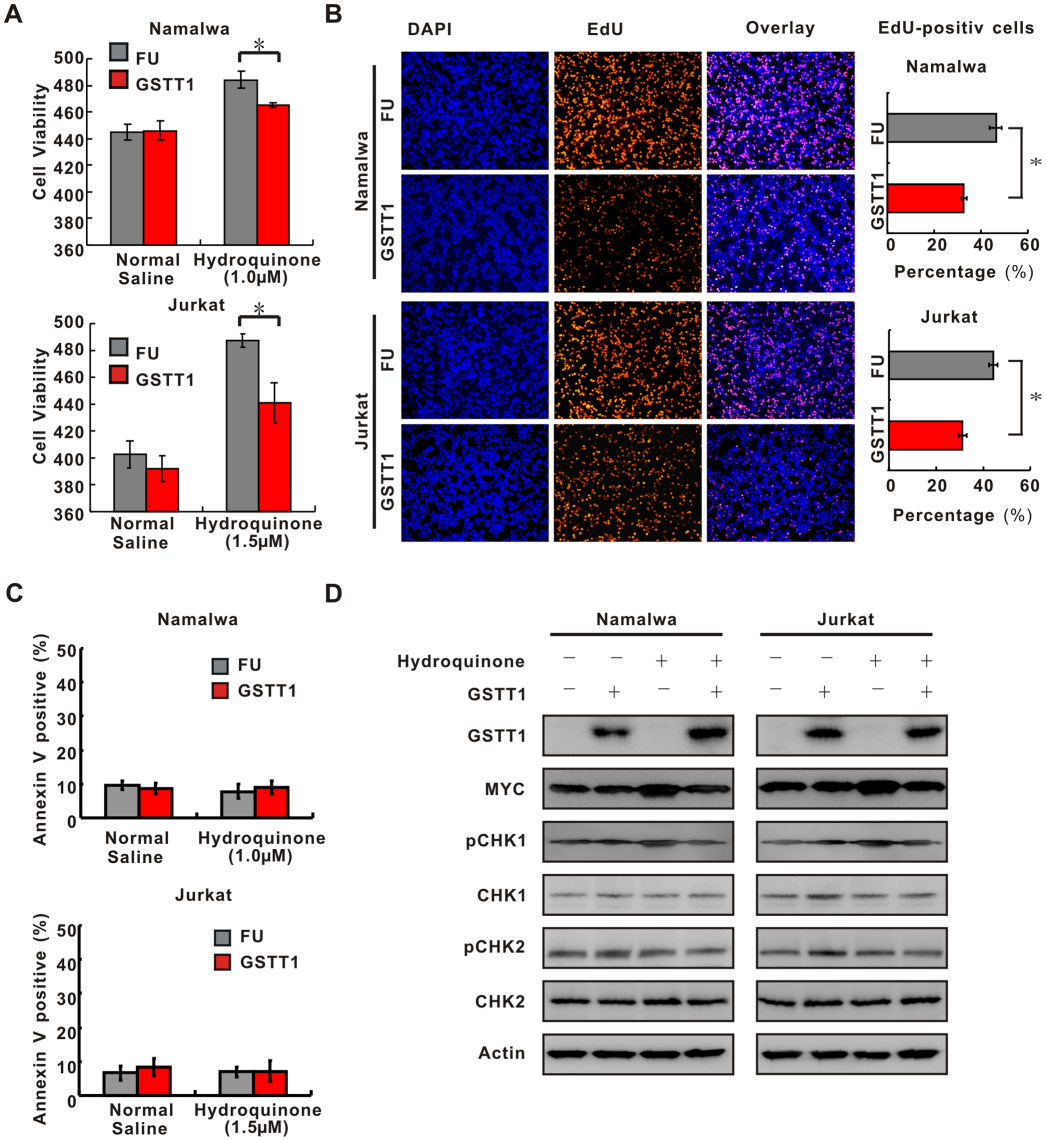
*GSTT1* expression inhibits PAH-mediated lymphoma cell proliferation. A: Effect of *GSTT1* expression on cell proliferation. 3×10^5^ cells treated with normal saline or Hydroquinone were seeded and cell number was counted at 48 h by typan blue. Data represent Mean±S.E. of densitometric values from three individual experiments. B: EdU assay of Namalwa and Jurkat cells treated with normal saline or Hydroquinone. C: Cell apoptosis analyzed by flow cytometry. Histography indicates Mean±S.E. from three individual experiments. D: Key proteins of DNA damage and cell cycle detected by Western blot in Namalwa and Jurkat cells with or without Hydroquione treatment. *P<0.05 comparing with the FU cells.

Upon treatment with normal saline, there is no difference of cell growth between the FU and GSTT1 groups. However, cell growth was enhanced in Hydroquinone-treated FU cells, which could be significantly reduced by ectopic expression of *GSTT1* (Namalwa, P = 0.045 and Jurkat, P = 0.043, respectively, Figure 4A). Cell proliferation was further determined by EdU assay. Comparing with the FU groups, EdU-positive cells in the GSTT1 groups were accordingly reduced (Namalwa, P = 0.043 and Jurkat, P = 0.039, respectively, Figure 4B). Cell cycle analysis showed a lower percentage of S-phase cells in the GSTT1 groups than in the FU groups (Namalwa, 17.5±0.52% vs 14.3±0.55%, P = 0.013, and Jurkat, 14.1±0.44% vs 11.8±0.54%, P = 0.032, respectively). As for cell apoptosis, neither Namalwa nor Jurkat cells showed obvious change in the percentage of ANX-V-positive cells between the FU and GSTT1 groups when treated with Hydroquinone (Figure 4C).

By Western blot, expressions of cell cycle regulation proteins were assessed with or without Hydroquinone treatment. In Hydroquinone-treated Namalwa and Jurkat cells, GSTT1 expression was consistent with downregulation of MYC, pCHK1 and nuclear 53 BP1, while pCHK2, total CHK1 and CHK2 levels remained constant (Figure 4D).

### GSTT1 Retards Tumor Formation of PAH-treated Lymphoma Cells

**Figure 5:**
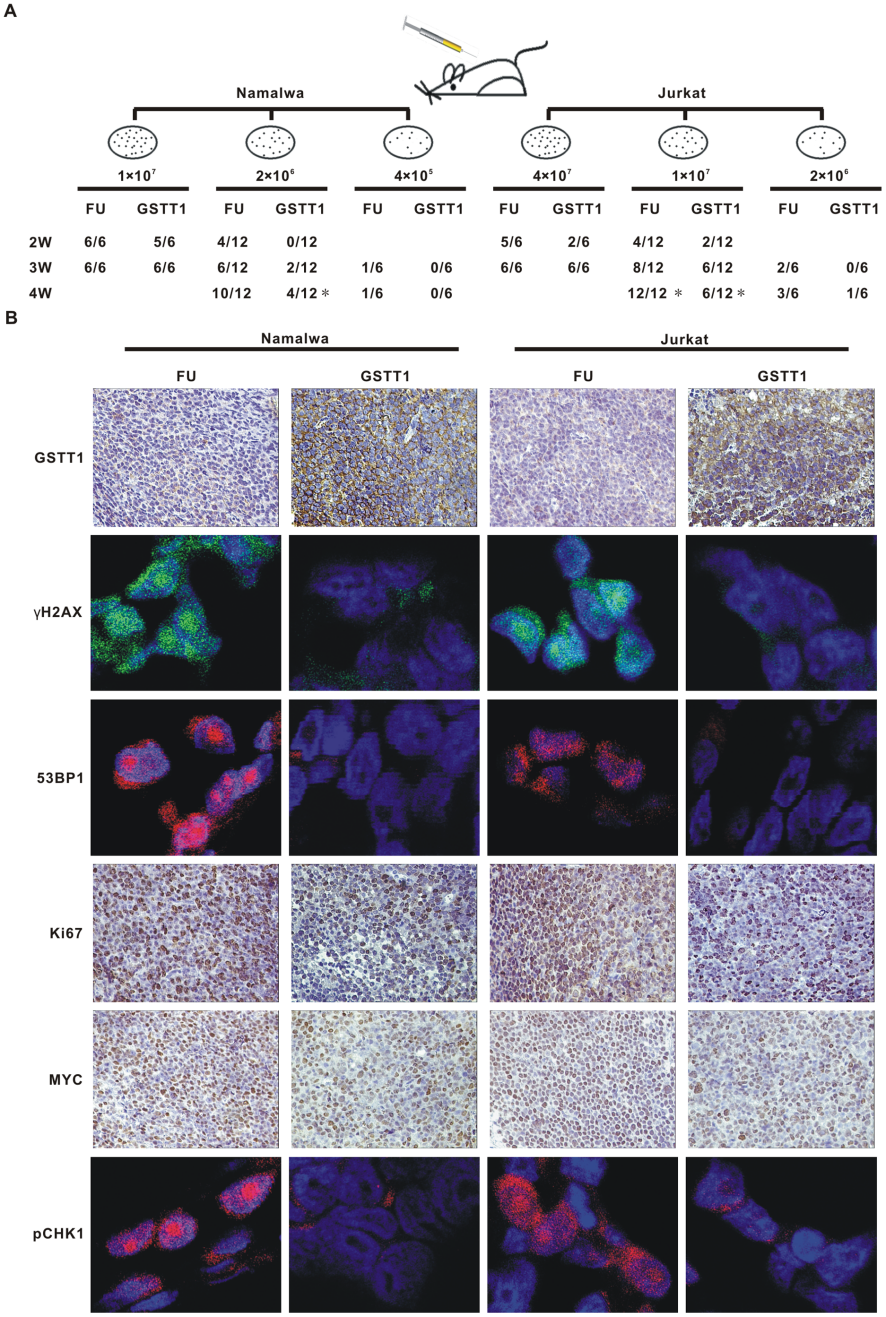
*GSTT1* expression reduces tumorogeneity of PAH-treated lymphoma cells. A: Tumor formation in nude mice. Indicated amount of Hydroquinone treated cells were injected subcutaneously and tumorigenicity was reported as numbers of tumors formed per numbers of mice injected. B: Expression of GSTT1, Ki67 and MYC detected in tumor tissues by immunohistochemistry assay, as well as pCHK1, γH2AX and 53 BP1 by immunofluorescence assay. *P<0.05 comparing with the FU cells.

To assess the carcinogenic potential in vivo, the FU and GSTT1 cells were treated with Hydroquinone as mentioned above and injected subcutaneously in nude mice (Figure 5A). Expression of *GSTT1* could significantly prolong the latency of tumor formation. Of note, 2×10^6^ Namalwa cells derived from the FU group could form tumors in 10 of 12 nude mice in 4 weeks. However, when 2×10^6^ cells from GSTT1 group were injected, only 4 tumors were found in 12 nude mice (P = 0.013). Similar results were obtained in Jurkat cells: tumor were formed in all mice at 4 weeks injected with 1×10^7^ FU cells, but only 6 of 12 mice in those treated with the same amount of GSTT1 cells (P = 0.014).

To search for *in situ* evidence of DNA damage and tumor cell proliferation, immunoflurescence assay of γH2AX, 53 BP1 and pCHK1, as well as immunohistochemistry assay of Ki67 and MYC were performed on mice tumor sections. In parallel with in vitro results, all markers were increased in the FU groups, compared with the GSTT1 groups following Hydroquinone treatment (Figure 5B).

### GSTT1 Knock-down Stimulates Lymphocyte Proliferation in PAH-treated Zebrafish

After searching the zebrafish genome data base (Zv9), two *GSTT1* genes were identified, referred as *gstt1a* and *gstt1b*, sharing part of nucleotide acids identical to human *GSTT1* gene. To confirm the evolutionary conservation of *GSTT1*, we investigated the distribution of genes located adjacent to the *GSTT1* locus in zebrafish and in human genomes. As shown in Figure S1A, 9 genes, along with *GSTT1*, define an approximately 840-kb genomic region on human chromosome 22, which is syntenic to the zebrafish *gstt1a* genomic locus on linkage group 8 and zebrafish *gstt1b* on linkage group 21. Thus, zebrafish *gstt1a* and *gstt1b* are evolutionarily conserved orthologs of human *GSTT1*.

Then the temporal and spatial expression patterns of *gstt1a* and *gstt1b* were examined in zebrafish embryos from 0.25 h to 120 hpf by WISH using digoxigenin-labeled antisense RNA probe. Strong signals were detected in the blastomeres of the two-cell stage and the shield period (6 h, Figure S1B), indicating that both *gstt1a* and *gstt1b* transcripts were maternally derived and played a role in early embryonic development. Although both genes exhibited similar patterns of expression during the early stage, differential expression was observed after 30 hpf: high levels of *gstt1a* transcripts were mostly restricted to the liver (2d), while *gstt1b* transcripts gradually disappeared after 30 hpf and was no longer observed after 3d (Figure S1B).

To further determine the function of GSTT1 on normal lymphocytes, morpholino was applied to efficiently block *gstt1a* and *gstt1b* gene expression in zebrafish. Injection of MO of *gstt1a* at the dosage of 8 ng per embryo, but not mismatch oligo control, was able to completely suppress the EGFP expression of co-injected capped *gstt1a*-EGFP mRNAs reporter (Figure S1C, left panels), indicating that the antisense RNA that could effectively silence gene expression. Similar results were obtained in *gstt1b* (Figure S1C, right panels).

Exposure of BaP, a carcinogenic PAH, was conducted at the concentration of 10 µg/L, based on previous experiments investigating the teratogenicity of PAH on zebrafish [14], [15]. Zebrafish embryos were microinjected with *gstt1a* and *gstt1b* morpholinos or 5 bp mismatch oligo controls, and treated with BaP from 24 hpf to 4 d. DMSO was used as the solvent control at a final concentration of 0.1%. At least 100 embryos were included in each group.

**Figure 6:**
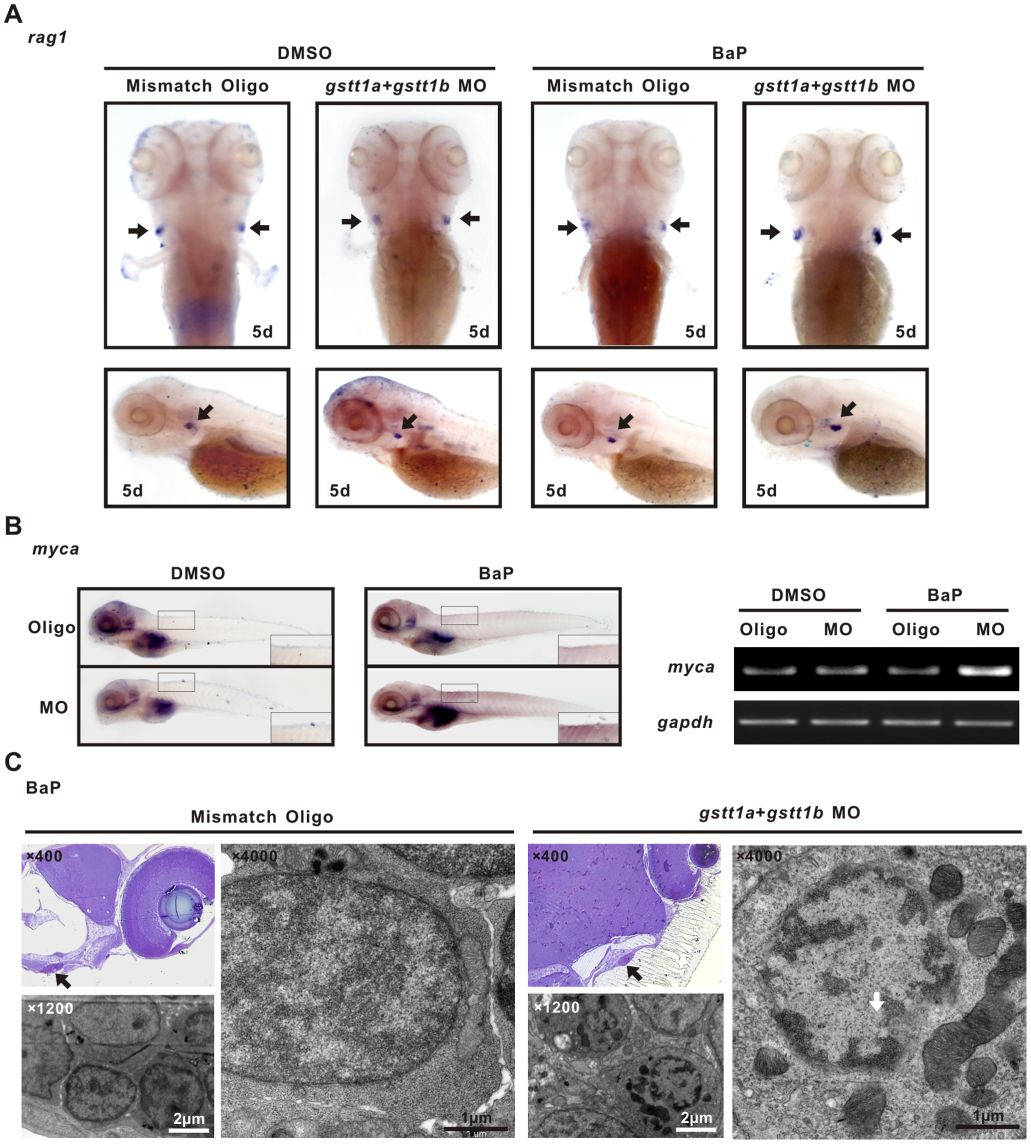
Knock-down of *gstt1a* and *gstt1b* promotes lymphocyte proliferation exposed to BaP. A; WISH images showed the *rag1* expression in the thymus (arrows) of differently treated 5 dpf embryos. B: In situ analysis of *myca* at 5 dpf. The morphants showed increased expression of *myca* in microinjected *gstt1a* and *gstt1b* morpholino exposed to BaP (Left panels), semi-quantitative PCR showed similar expression pattern in embyos (Right panels). C: Ultrastructure of thymic lymphocytes from 5 dpf larvae exposed to BaP. Images represent results from three independent experiments and each group contains 30 morphants.

Comparing with those injected with mismatch oligos, *gstt1a* and *gstt1b* silenced embryos showed an increase of the *rag1* signal in the thymus under exposure to BaP, as detected by WISH (Figure 6A, right panels). No significant difference of the *rag1* signal was observed in embryos treated with DMSO (Figure 6A, left panels).

To confirm that MYC activation is also involved in PAH-associated stimulation of lymphocyte proliferation in zebrafsh, the expression of *myca*, zebrafish homologue of human MYC, was assessed in BaP-treated embryos at 5 d. In accordance with *rag1* expression, *myca* expression was significantly higher in embryo injected with *gstt1a* and *gstt1b* morpholinos than those injected with mismatched oligos by WISH (Figure 6B, left panels) and by semi-quantitative PCR (Figure 6B, right panels).

The structure of the thymus in 5 d-embryos was examined by transmission electron microscopy (Figure 6C). Ultrastructural analysis of both cells (microinjected with *gstt1a* and *gstt1b* morpholino or oligo control) revealed no sign of apoptosis. At a higher magnification, abundant euchromatin and robust mitochondrias were observed in BaP-treat *gstt1*-silenced lymphocytes, possibly related to a hyper-proliferative status of the cells.

Therefore, in addition to lymphoma cells, inactivation of *GSTT1* was able to confer a proliferative advantage induced by PAH in their normal counterparts.

## Discussion

Although environmental factors are proven relevant for tumorogenesis, few genetic variations with confirmed associations to date and the difficulty in accurately assessing exposures are main challenges to evaluate the interaction between gene and environment in cancer [16]. Our study provided evidence that genetic polymorphisms in *GSTT1* gene, both in neoplastic and non-neoplastic situation, could modulate the response of the lymphocytes to the major component of environmental pollutants PAH and might link to lymphoma development.

The *GSTT1*-null genotype was more prevalent in lymphoma patients than in normal controls, conferring approximately a 1.7-fold increase in the risk of lymphoma. This coincides with epidemiological studies from Western [4], African [5], and other Asian countries [6], although the incidence of *GSTT1* deletion varied among the geographical areas. The genetic polymorphism was associated with loss of gene and protein expression, indicating that *GSTT1* is functionally impaired in lymphoma.

It is previously reported that deletions in the *GSTT1* gene contribute to individual susceptibility to PAH-induced DNA damage and carcinogenesis [17], [18]. Our study further showed in lymphoma that introduction of GSTT1 to *GSTT1*-negative tumor cells is associated with increased DNA stability and repair of oxidative DNA damage in response to PAH. These circumstances fit to the idea that GSTT1 was involved in the susceptibility of individuals to PAH-induced DNA damage, loss of which favoring the accumulation of cytogenetic aberrations and thereby leading to the initiation of lymphoma. Therefore, genetic polymorphisms in detoxification enzymes might account for individual variation in lymphoma risk and should be considered in a more complex scenario involving the gene-environment interactions.

GSTT1 can modulate multiple cellular processes, including cell proliferation and cell death [19]. Among the lymphoma patients with the history of PAH exposure, instead of cell apoptosis, the *GSTT1*-deleting cases displayed a genomic profile of cell cycle progression, referring dysregulation of cell proliferation as the major target of *GSTT1* deletions in lymphoma. Experimentally, in *GSTT1*-negative lymphoma cells, expression of *GSTT1* significantly prohibited PAH to enhance tumor cell growth, with tumor aggressiveness accordingly decreased in murine models. These data thus suggested a potential role for GSTT1 in protecting against lymphoma cell proliferation provoked by PAH.

MYC is essential for cell proliferation [20] and is a proto-oncogene frequently upregulated in lymphoma [21]. More importantly, MYC itself can localize onto sites of active DNA replication and directly controls S-phase cell progression [22]. MYC was activated following PAH treatment in our study, particularly in *GSTT1*-negative lymphoma cells, corresponding to increased S-phase cells, enhanced cell proliferation and in vivo tumorigenicity, indicative the possible involvement of MYC on PAH-associated cell cycle dynamics and lymphoma progression in GSTT1-null status.

The MYC-induced DNA damage response acts as a double-edged sword in tumor progression. CHK1 and CHK2 are key factors involved in the replication stress response and controlled by MYC [23]. Indeed, activation of CHK1 is essential for tumor maintenance, while CHK2 activity constitutes a barrier to malignant transformation. As previously reported in Eμ-myc lymphoma models, tumor cells present increased levels of CHK1 phosphorylation, in turn limits MYC-induced apoptosis. In the clinical setting, lymphoma patients exhibit a striking correlation between high levels of MYC and CHK1 [24]. This was also proven by us in PAH-treated lymphoma cells, where MYC might ensure proliferative advantage through selectively activating CHK1. Recent reports have identified MYC-positive lymphoma as a subtype with poor disease prognosis [25], even resistant to high-dose chemotherapy [26] and newly developed bio-therapeutic agent [27]. Since MYC is difficult to be targeted directly, CHK1 inhibitors could thus become attractive candidates for therapeutic intervention on MYC-driven malignancies and warrant further investigation.

Genetic factors that impair DNA repair can increase the likelihood of pre-neoplastic changes [28]. This is particularly obvious when environmental factors were existed, as a previous report showing that the t(14;18)-positive clones are prominent in individuals exposed to pesticides and correlated with a higher risk of t(14;18) lymphoma [29]. In addition to lymphoma cells and murine xenograft models, we used zebrafish as an animal model to verify the cooperative effect of the genetic and environmental factor on their normal counterparts. In *GSTT1*-knock-down zebrafish, although may not initially be lethal, genomic lesions in lymphocytes could be modulated by PAH that promote lymphocyte proliferation and MYC upregulation, which could eventually link to malignant transformation of lymphoma.

## Supporting Information

Figure S1 ***GSTT1*** **is evolutionarily conserved and expressed ubiquitously during embryonic development.** A: Comparison of the syntenic relationship of the zebrafish *gstt1* genes with the human orthologue. Orthologous *gstt1* genes symbols were in bold. Other pairs of duplicated genes (e.g. *mmp11a* and *mmp11b*) on zebrafish and the human (e.g. *MMP11*) orthologue are underlined. Hs_Chr, Homo sapiens chromosome, Dr_LG, Danio rerio linkage group, Mb, megabase. B: Expression of zebrafish *gstt1a* and *gstt1b* in wild-type AB strain embryo during embryonic development. C: Efficiency validation of EGFP reporter expression by *gstt1a* morpholino and *gstt1b* morpholino. Images represent the typical outcome of three independent experiments and each group contains 30 morphants.
(TIF)
