# Supplementary figures and images for: GSTT1 Deletion Is Related to Polycyclic Aromatic Hydrocarbons-Induced DNA Damage and Lymphoma Progression
Source: PLoS One. 2014 Feb 20;9(2):e89302. doi: 10.1371/journal.pone.0089302 (PMC3930712; doi:10.1371/journal.pone.0089302)

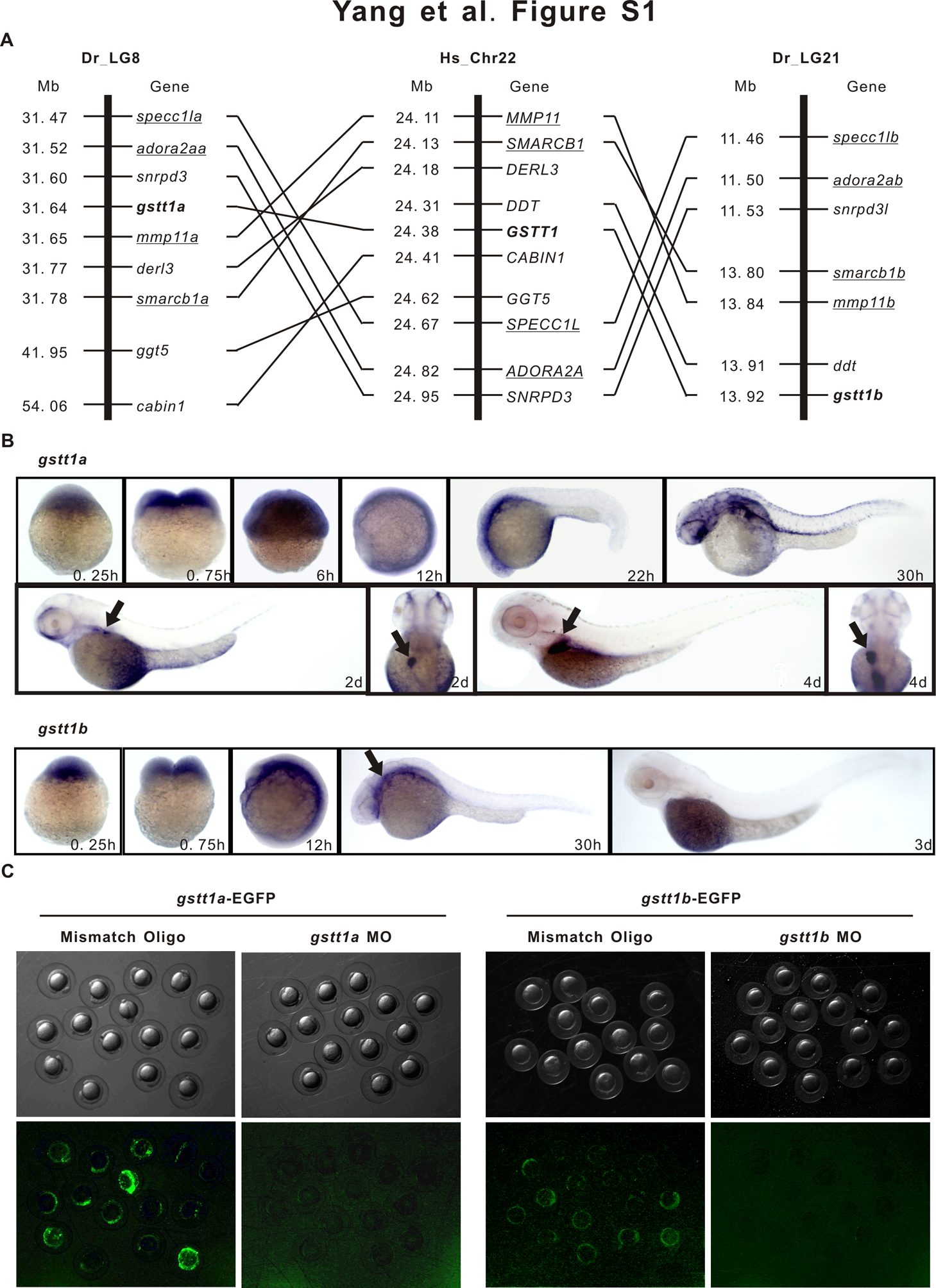

Supplement: Figure S1 — GSTT1 is evolutionarily conserved and expressed ubiquitously during embryonic development. A: Comparison of the syntenic relationship of the zebrafish gstt1 genes with the human orthologue. Orthologous gstt1 genes symbols were in bold. Other pairs of duplicated genes (e.g. mmp11a and mmp11b) on zebrafish and the human (e.g. MMP11) orthologue are underlined. Hs_Chr, Homo sapiens chromosome, Dr_LG, Danio rerio linkage group, Mb, megabase. B: Expression of zebrafish gstt1a and gstt1b in wild-type AB strain embryo during embryonic development. C: Efficiency validation of EGFP reporter expression by gstt1a morpholino and gstt1b morpholino. Images represent the typical outcome of three independent experiments and each group contains 30 morphants. (TIF) [file pone.0089302.s001.tif]
